# Health service use and costs associated with fluoroquinolone‐related tendon injuries

**DOI:** 10.1002/prp2.796

**Published:** 2021-06-04

**Authors:** Laura S. M. Kuula, Janne T. Backman, Marja L. Blom

**Affiliations:** ^1^ Faculty of Pharmacy University of Helsinki Helsinki Finland; ^2^ Individualized Drug Therapy Research Program Faculty of Medicine Department of Clinical Pharmacology University of Helsinki University of Helsinki and Helsinki University Hospital Helsinki Finland

**Keywords:** adverse drug reactions, antibiotics, costs, tendons

## Abstract

The aim of this study was to assess costs and health service use associated with tendon injuries after the use of fluoroquinolone antimicrobials in Finland during 2002–2012. This retrospective observational study included data from the Finnish Pharmaceutical Insurance Pool's pharmaceutical injury claims. In total, 145 compensated claimants aged ≥18 years presenting tendon injuries after the use of fluoroquinolones (FQs) were included in the study. Outcomes of interest were the number of outpatient visits to primary, secondary, tertiary, and private healthcare services, hospital days, rehabilitation and their costs. Regression models were used to analyze the impact of patient characteristics on hospital days, as well as the relationship between patient characteristics and tendon ruptures. Direct costs of a tendon injury averaged 14,800€ and indirect costs were estimated to be 9,077€ for employed claimants. Fifty‐one percent of the claimants were hospitalized, with an average duration of 21 days. Hospitalization was the costliest form of health service use with an average of 9,915€ per hospital episode. Hospital days and direct costs increased with the severity of the injury. Tendon ruptures, in particular bilateral ruptures, required substantially more hospital days and their direct costs were significantly higher than those of uncomplicated tendinitis. Concurrent use of oral corticosteroids and increasing age were associated with a higher likelihood of tendon ruptures. Although rare, FQ‐related tendon injuries can result in considerable costs and health service use. Medical staff should remain vigilant when prescribing FQs, especially in groups at increased risk for tendon injuries.

## Graphical abstract

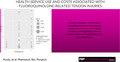

**Abbreviations**
AEs: adverse events
EMA: European Medicines Agency
FDA: Food and Drugs Administration
FQs: Fluoroquinolones

## 1 INTRODUCTION

Fluoroquinolones (FQs) are a class of broad‐spectrum antimicrobials that have been in wide clinical use since the 1980 and are used to treat bacterial infections including genitourinary, respiratory, gastrointestinal, skin, and soft tissue infections.^1^, ^2^ Although FQs are generally well tolerated, they have been associated with serious adverse events (AEs), such as tendon injuries, aortic wall injuries, and neuropathies. Consequently, the European Medicines Agency (EMA) and the U.S. Food and Drugs Administration (FDA) have recommended several restrictions on their use.^3^, ^4^, ^5^ Tendon ruptures and tendinitis are rare but debilitating and possibly costly AEs that have been strongly associated with the use of FQs. According to a recently published cohort and nested case–control study, the excess risk of a tendon rupture due to fluoroquinolones is about 3.7 cases per 10,000 person‐years.^6^ Signs of tendinitis include tendon swelling, irritation, and moderate‐to‐severe pain. Tendon ruptures, on the other hand, usually cause severe pain, weakness, and deformity of the affected tendon. Tendon injuries are treated with immobilization and/or corrective surgery.^7^ Both tendinitis and tendon ruptures can either heal completely or cause permanent damage to the tendon. The treatment of these serious AEs often requires both sick leave and health service use, which, in turn, cause potentially significant costs for patients and society. Previous research has shown that assessments of FQ‐related AE costs are scarce even though hospitalization is frequently required to treat AEs.^8^

The specific molecular pathway causing FQ‐related tendon injuries remains unidentified. The biological antimicrobial mechanism of FQs is based on targeting bacterial DNA gyrase (DNA topoisomerase),^9^ and accordingly, a possible mechanism of AE is that FQs not only target bacterial DNA but also human DNA. A recent study has proposed that most FQ‐related AEs could be explained by mitochondrial DNA effects due to the loss or inhibition of type II topoisomerase Top2β.^10^ In addition, there is evidence of a connection between FQs and other collagen‐associated AEs, such as aortic aneurysms, aortic dissections, and retinal detachments, in addition to tendon injuries.^11^ Previous research has shown that concurrent use of corticosteroids, an age of over 60 years, and chronic kidney disease are the risk factors for developing FQ‐related tendon injuries.^6^, ^12^

The aim of this study was to assess costs and health service use associated with tendon injuries relating to FQ antimicrobial use in Finland during 2002–2012. Although FQ‐related tendon injuries were first reported in 1983,^13^ as far as we know, the costs associated with them have previously not been studied.

## 2 MATERIALS AND METHODS

This retrospective observational study analyzed data obtained from the Finnish Pharmaceutical Insurance Pool's pharmaceutical injury claims. In Finland, patients, who suffer a pharmaceutical injury are entitled to compensation, if a causality can be detected between the pharmaceutical and the injury. A pharmaceutical injury is a legal term defined as any bodily illness or injury or a psychiatric disease likely to result from a pharmaceutical taken by the injured party.^14^ Pharmaceutical injury compensations are determined by applying the provisions contained in Finland's Tort Liability Act and the guidelines issued by the Traffic Accident Board.

The costs of FQ‐related tendon injuries were evaluated from the Insurance Pool's compensated insurance claims from 2002 to 2012. FQ‐related tendon injury costs were borne by the Social Insurance Institution of Finland, municipalities, employers, patients, and the Finnish Pharmaceutical Insurance Pool. Of these payers, the latter is a secondary insurance system and only compensates patient costs relating to pharmaceutical injuries, which are not covered by other institutions. These costs include excess healthcare and travel costs, temporary incapacity compensation (pain and suffering), permanent functional and cosmetic incapacity, loss of income, and loss of life compensation. In order to receive compensation, the pharmaceutical injury claim must adhere to the terms and conditions of the Finnish Pharmaceutical Insurance Pool. For example, the pharmaceutical injury claim must be submitted within 3 years of the claimant becoming aware of the injury or no later than 10 years after discontinuing taking the pharmaceutical. Additionally, no compensation is rewarded if the pharmaceutical injury is regarded as tolerable in relation to the nature and severity of the illness being treated.^14^ Health service use in Finland consists of visits to public, private, and occupational healthcare. Finland's publicly funded healthcare system is divided into three levels and consists of primary health centers, secondary central hospitals, and tertiary university hospitals. The healthcare system is organized by municipalities and divided into 21 larger hospital districts and five university hospitals. The private healthcare sector is much smaller and less frequently used. Additionally, employers are obliged to organize and fund occupational healthcare for employees. In this study, FQ‐related AE costs were examined from a societal perspective as all AE‐related costs, regardless of whom they fall on. FQ‐related AE costs were divided into direct and indirect societal costs.

Direct societal costs of a FQ‐related tendon injury episode were the primary outcome variable of the study, while other outcomes included the amount of outpatient visits to primary and private healthcare, visits to secondary and tertiary care, hospital days, rehabilitation and their costs in addition to travel costs. Besides direct tangible costs, intangible costs consisting of temporary and permanent incapacity compensation and time costs were quantified and added to direct costs. Outpatient visits and hospitalizations were valued with the unit costs of social and healthcare services in Finland.^15^, ^16^, ^17^ Travel costs were quantified by calculating the distance to the used health service providers and multiplied by the Finnish Tax Administrations annually defined kilometer allowance.^18^ Time costs comprised the time spent travelling to the healthcare facility, the time receiving treatment and waiting time. The estimate represents the age‐specific value of lost time or opportunity costs. The approximations of time costs are based on Finnish estimates,^19^ which were adjusted to 2017 euro with the index of wage and salary earnings.

Indirect costs of the employed claimants were estimated according to the friction cost approach.^20^ Indirect costs accounted for the loss of productivity due to FQ‐related tendon injuries during a friction period when a substituting employee was searched to replace the injured employee. The average friction period in hours was multiplied by the average hourly loss productivity estimate based on the Statistics Finland's average earnings and average social costs paid by the employer.^21^ Average Finnish earnings were chosen due to lack of salary information in the data. The friction period was estimated from Statistics Finland's Employment service statistics^22^ and following Tan et al.,^23^ an additional 4‐week period was added to estimate the time employers require to decide to open a vacancy. Due to elasticity between labor time and labor productivity, we applied a 0.8 elasticity measurement to indirect costs, as suggested by Koopmanschap et al.^20^

Previous studies have shown hospitalization to be a costly form of health service use.^24^, ^25^ As there was a high occurrence of no hospital days in the dataset, a two‐part model was built with R (version 3.6.2.) to predict hospitalization and the length of hospital stay. Additionally, two Generalized linear models (glm) were built to estimate the association of tendon ruptures and specifically bilateral ruptures with patient characteristics. Several subgroup analyses were done to various subsets of claimants, so that their age, sex, use of oral corticosteroids, the number of co‐morbidities or the fluoroquinolone derivative used could be taken into account. All costs were converted to 2017 euro.

## 3 RESULTS

### 3.1 Claimants

**FIGURE 1:**
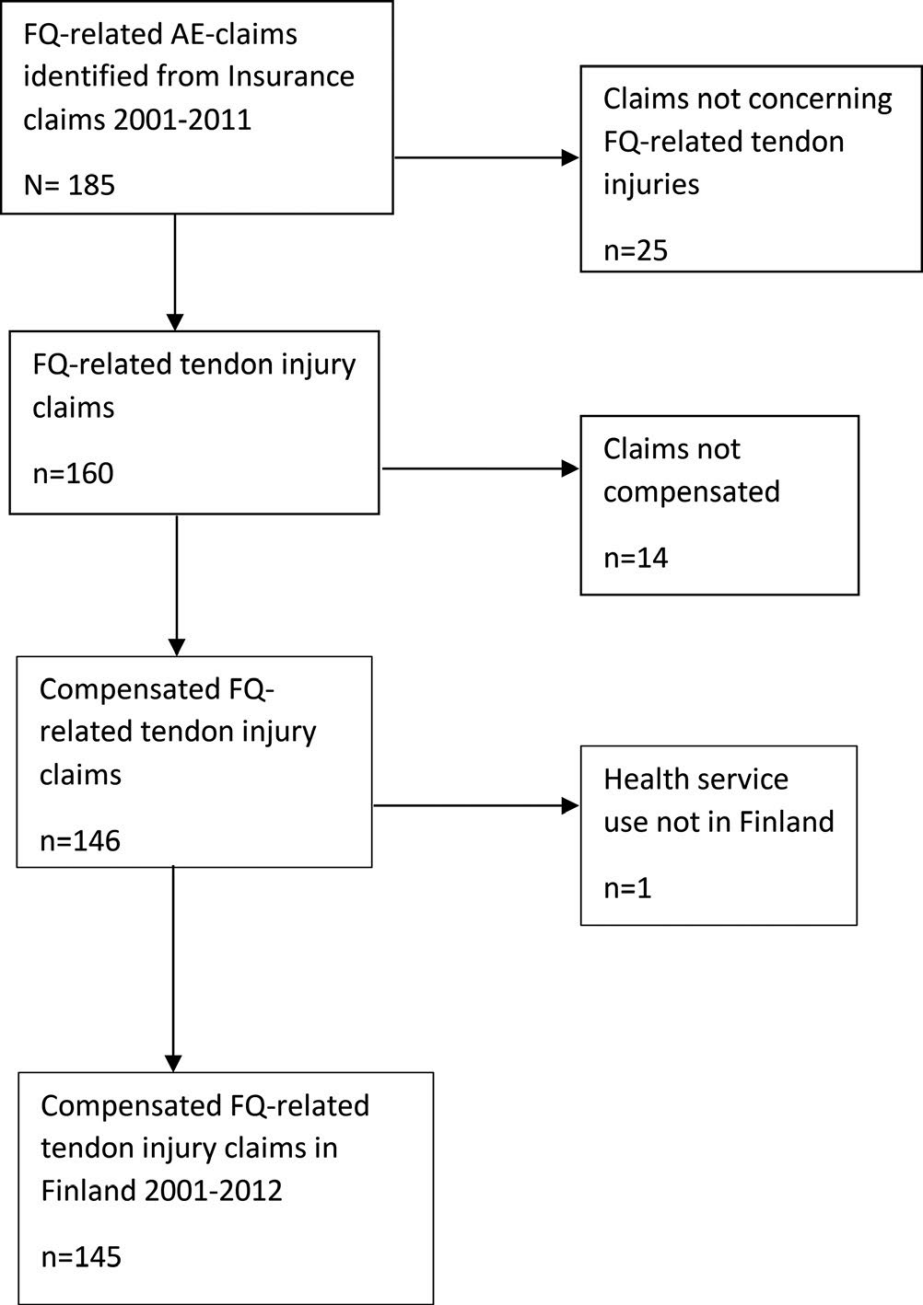
Study eligibility and flow diagram of the selection process of claimants

**FIGURE 2:**
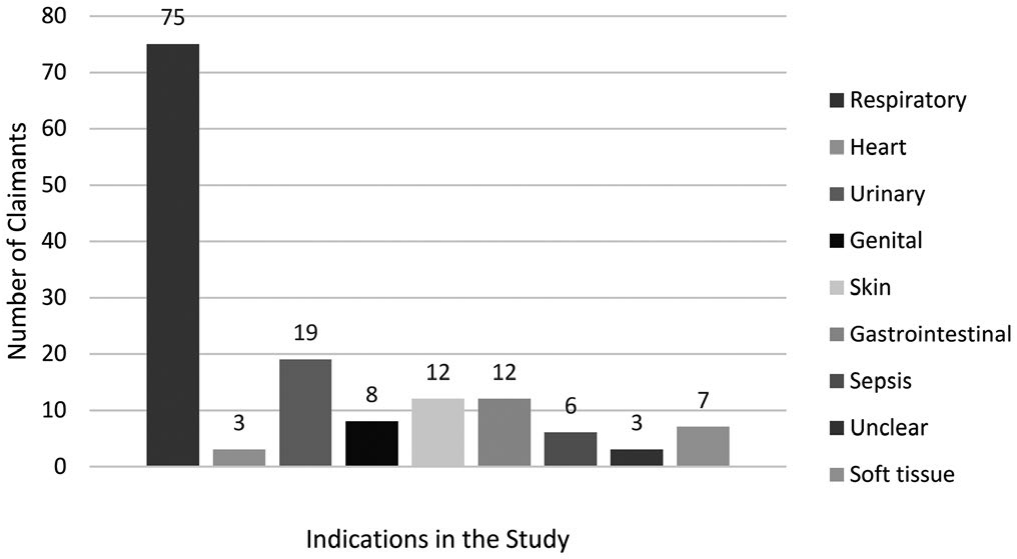
Indications for FQ use in the study

In total, 145 compensated claimants aged ≥18 years presenting tendon injuries classified as tendinitis (*n* = 52) or tendon ruptures (*n* = 93) after the use of fluoroquinolones were included in the study, after excluding 25 + 14 Figure 1. Sixteen percent of the included claimants (*n* = 23) suffered bilateral ruptures. Ninety‐nine of the claimants were men and 46 were women and their average age was 66.8 years (range 21–87). Tendon injuries were reported in connection with all FQs currently on the market in Finland. In all, 121 cases (83%) were associated with levofloxacin, 13 (9%) with ciprofloxacin, 7 (5%) with norfloxacin, 2 (1%) with ofloxacin, and 2 (1%) with moxifloxacin. The majority of the claimants (52%) were treated for respiratory infections Figure 2. Co‐morbidities were common, and only 10% of the claimants did not have a pre‐existing health condition, while 45% had been diagnosed with a chronic lung disease. A total of eight claimants died during the compensation claim process. Claims were filed from all Finnish hospital districts. In this study, 97% of the injuries were associated with Achilles tendons. Other affected tendons included those of the quadriceps and tibialis posterior muscles and the rotator cuff. Tendon injuries occurred on average within 3 weeks of the first dose of FQs (median 10 days) but ranged between 24 h and 20 years.

### 3.2 Direct costs and health service use

The estimated average direct societal cost of a FQ‐related tendon injury episode per claimant, including incapacity compensation, was 14,800€. Without incapacity compensation, a FQ‐related tendon injury amounted to 8744€. The total direct costs associated with FQ‐related tendon injuries among claimants were 2,146,057€. Hospitalization was the costliest form of health service use. The claimants frequently visited a primary care facility (331 visits) and rehabilitation, such as physical and lymphatic drainage therapy (686 visits). The averages of health service use and costs are presented in Table 1. Of all the claimants, 74 (51%) were evaluated to have permanent tendon injuries due to FQ use, and 74 (51%) claimants were hospitalized, with an average duration of hospitalization of 21 days (range 1–87). Tendon ruptures (*n* = 93) were most often treated conservatively (equinus cast for 3–9 weeks), while 33 (23%) claimants’ tendon injuries were treated with surgery.

**TABLE 1:** Health service use and direct societal costs associated with FQ‐related tendon injuries

	Hospitalization	Primary healthcare^a^	Secondary healthcare	Tertiary healthcare	Private healthcare	Rehabilitation	Travel costs	Time Costs	Pharmaceutical injury compensation	Incapacity compensation	Total direct costs
Claimants affected (n)	74	88	79	41	39	56	128	145	145	145	145
Average of days or visits (n)	20.61	3.7	3.98	3.98	4.1	12.25					
Median of days or visits (n)	8	2	3	4	3	10					
Range of days or visits (n)	0–87	0–33	0–14	0–10	0–14	0–80					
Total days or visits (n)	1525	331	315	163	161	686					
Average costs for claimants using €	9914.70	267.54	875.20	867.30	704.20	755.40	276.20				
Median €	5036.50	160.29	599.90	720.70	529.05	562.31	121.60	131.54	5081.30	5030.30	89945.00
Range €	0.00–83,554.60	0.00–1273.10	0.00–2434.70	0.00–1811.30	0.00–3079.40	0.00–3492.10	0.00–3421.70	6.60–3519.90	120.90–199,712.80	0.00–23,777.66	1198.10–201,063.70
Average for whole study population €	5059.90	162.40	247.50	245.20	189.40	291.70	243.84	292.50	8283.00	6056.30	14,800.00
Total costs €	733,685.90	23,543.30	59,748.80	35,559.40	27,462.80	42,302.30	35,357.10	42,417.00	1,201,031.00	878,162.80	2,146,057.40

Note

The data are from the 145 claimants who received compensation based on the Finnish Pharmaceutical Insurance Pool's pharmaceutical injury claims 2002–2012.

^a^ Includes visits to primary health clinics and occupational healthcare.

### 3.3 Indirect costs

Fourteen percent (20) of the claimants were employed at the time of their FQ‐related AEs and the Insurance Pool compensated losses of income due to FQ‐related tendon injuries in the case of six claimants (aged 39–66 years), which amounted to an average cost of almost 50,000€. In one case, the tendon injury led to permanent disability pension payments. Sick leave data were available for 16 claimants, whose sick leave lasted for an average of 78 days (range 1–331 days).

The average productive work time of an employee in Finland is estimated to be 7.25 h per day, 5 days a week. The estimated friction period was thus 8 + 4 weeks, which amounted to 12 × 5 × 7.25 productive hours, that is 435 h. The average loss of productivity cost estimate was 26.08€/hour. Consequently, the indirect costs of a friction period would have amounted to 11,346€. When these costs were multiplied by the elasticity measurement 0.8, indirect costs of a FQ‐related tendon injury for an employed person amounted to 9077€ per episode.

### 3.4 Statistical analyses

Dichotomous and continuous independent variables were selected to determine the impact of known risk factors, AE severity and AE year on hospitalization. As approximately half of the claimants had no hospital days and the data exhibited over dispersion, a two‐part model was built to combine the presence of excess zeros and the negative binomial distribution of counts in the data (Table 2). The first part, the binomial logit model, predicted the probability of no hospitalization for all claimants (*n* = 145). The second part, a zero‐truncated negative binomial model, predicted the length of hospital stay for those claimants (*n* = 74) who had at least one day of hospitalization. Negative and positive coefficient estimate variation is explained by the logit model modeling the probability of zero and the zero‐truncated modeling a positive count. The results of the two‐part model indicated that claimants who had a tendon rupture had significantly smaller odds to avoid hospitalization that those with tendinitis (logit model OR = 0.3044, *p* = .00316) as well as a 4.063 time increase in length of hospital stay (zero‐truncated model IRR = 4.063, *p* = .000053), given that the other variables are held constant in the model. Additionally, advancing age and having co‐morbidities made claimants more likely to have longer hospital days or less likely to avoid hospitalization, respectively. The prescribed FQ, use of oral steroids, or the claimant's gender had no significant influence on hospitalization in this model. Norfloxacin was used as a reference variable in categorizing FQs. Changing the reference FQ did not alter the results.

**TABLE 2:** Analysis of patient characteristics associated with no hospitalization (logit model) and length of hospital stay (zero‐truncated model) in the 145 claimants who received compensation from the Finnish Pharmaceutical Insurance Pool, 2001–2012

Variable	Logit binomial			Zero‐truncated negative binomial		
	Estimate ± standard error	*p*‐value	OR (95% CI)	Estimate ± standard error	*p*‐value	IRR (95% CI)
AE year	0.140 ± 0.072	0.05139	1.1497 (1.001–1.3283)	−0.144 ± 0.056	NA	0.870 (0.78–0.97)
Male gender (1)	−0.047 ± 0.447	0.91636	0.954 (0.3958–2.3069)	0.119 ± 0.331	0.718	1.13 (0.59–2.16)
Age	0.015 ± 0.017	0.38930	1.015 (0.9809–1.0505)	0.048 ± 0.014	0.00048	1.05 (1.02–1.08)
Use of oral steroids (1)	−0.381 ± 0.386	0.32268	0.689 (0.3193–1.457)	0.141 ± 0.282	0.617	1.15 (0.66–2.00)
Use of levofloxacin (1)	−0.713 ± 0.958	0.45662	0.4903 (0.059–2.94)	−0.782 ± 0.944	0.407	0.458 (0.072–2.91)
Use of ciprofloxacin (1)	−0.731 ± 1.112	0.51077	0.4813 (0.0454–4.002)	−0.355 ± 1.009	0.725	0.7013 (0.097–5.07)
Use of moxifloxacin (1)	−0.876 ± 1.807	0.62761	0.4163 (0.0087–17.975)	−11.268 ± 32.561	0.729	0.000013 (2.45e‐33‐6.65e+22)

Use of norfloxacin	Reference	Reference	Reference	Reference	Reference	Reference
Use of ofloxacin (1)	−0.001 ± 1.737	0.99953	0.999 (0.0241–39.451)	−1.08785 ± 1.477	0.461	0.337 (0.0186–6.093)
Number of co‐morbidities	−0.379 ± 0.173	0.02900	0.6847 (0.4804–0.9517)	0.14277 ± 0.114	0.211	1.15 (0.922–1.442)
Ruptured tendon (1)	−1.189 ± 0.403	0.00316	0.3044 (0.1353–0.6618)	1.40201 ± 0.346	0.000053	4.063 (2.059–8.017)
Intercept (a)	−278.695 ± 143.6	0.05236	9e‐122 (2e‐247‐0.1764)	288.358 ± 112.82	0.011	1.707587e+125 (1.572627e+29‐1.854130e+221)
Intercept (b)				−0.11977 ± 0.245	0.625	0.871 (0.549–1.434)

Note

Negative coefficient estimates indicate inverse relationship between predictors and outcomes.

Abbreviations: CI, confidence interval; IRR, incidence rate ratio; OR, odds ratio.

**FIGURE 3:**
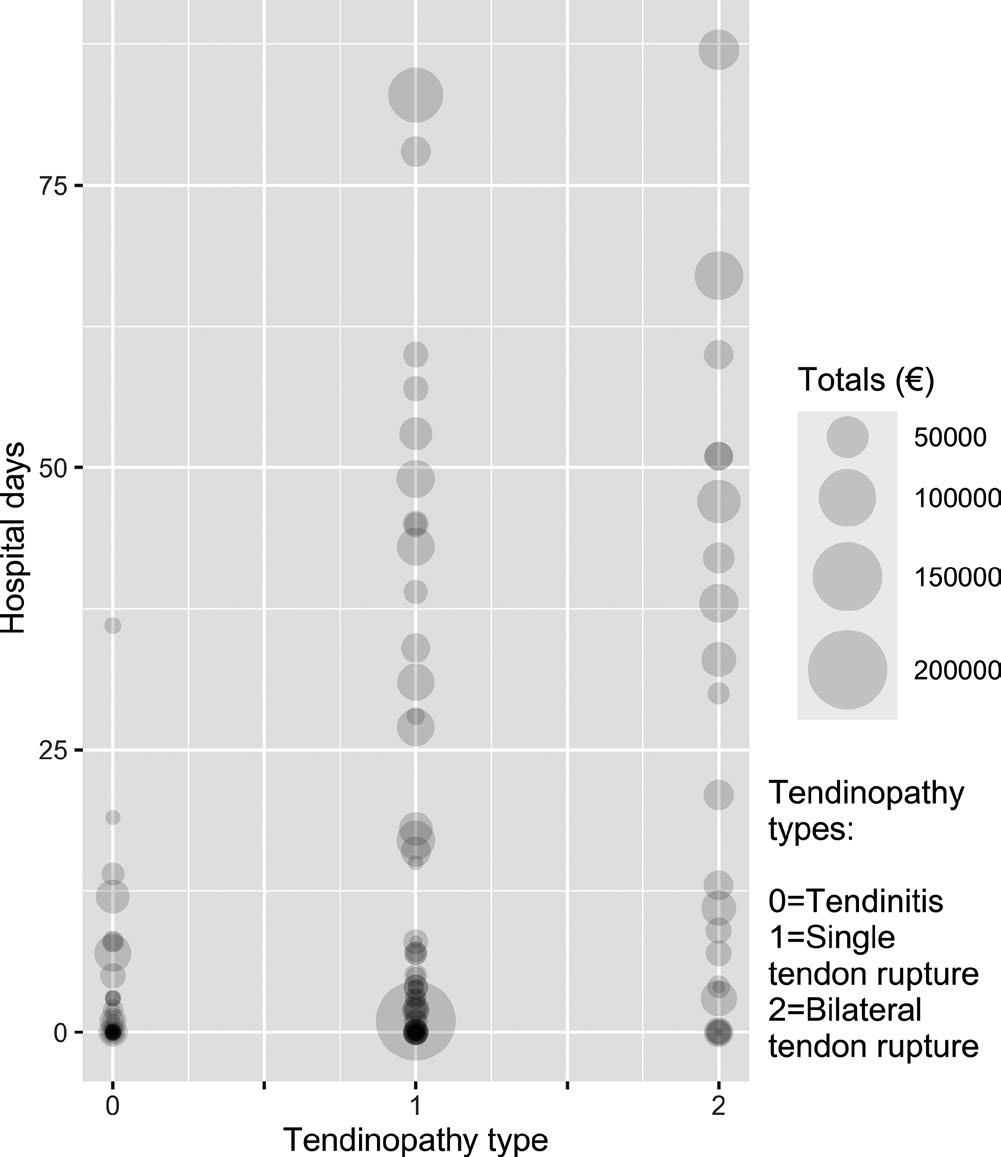
Impact of tendinitis and tendon rupture on hospital days and total direct costs in the 145 claimants who received compensation based on the Finnish Pharmaceutical Insurance Pool's pharmaceutical injury claims 2002–2012

Subgroup analyses were consistent with the results of the above models. Claimants with tendon ruptures required a mean of 15 days in hospital, and their total direct costs amounted to an average of 19,183€, whereas claimants with tendinitis were hospitalized less frequently, for an average of 2 days and with an average direct cost of 6963€. Bilateral tendon ruptures (*n* = 23) were the costliest subgroup. These claimants required hospitalization for an average of 25 days and their total direct costs averaged 25,731€. There were no substantial differences between males and females, different ages, treatment options, or AE years. However, claimants with co‐morbidities had on average 11 hospital days in comparison to healthy claimants who had only 2 hospital days. Figure 3 provides an illustration of the impact of tendinitis, in addition to single and bilateral tendon rupture on the amount of hospital days and total direct costs.

Glm logit models predicting tendon ruptures among the claimants showed that advancing age slightly and taking oral corticosteroids more prominently increased the odds of suffering a tendon rupture (OR = 1.035 and OR = 2.55, respectively) (Table 3). The impact of concurrent oral steroid use was more substantial among claimants with bilateral tendon ruptures (OR = 3.98, *p* = .00808) (Table 4). The number of co‐morbidities, and again, the prescribed FQs, AE year, and claimant gender were not statistically significant in these models.

**TABLE 3:** Analysis of patient characteristics’ association with tendon ruptures in the 145 claimants who received compensation based on the Finnish Pharmaceutical Insurance Pool's pharmaceutical injury claims 2002–2012

Variable	Estimate ± standard error	*p*‐value	OR (95% CI)
AE year	−0.0732 ± 0.0716	0.3067	0.929 (0.806–1.07)
Male gender	0.52851 ± 0.4399	0.2296	1.67 (0.715–4.05)
Age	0.03504 ± 0.0172	0.0421	1.035 (1.002–1.072)
Use of oral steroids	0.93709 ± 0.4125	0.0231	2.55 (1.16–5.89)
Use of levofloxacin	−0.4227 ± 0.9344	0.6510	0.655 (0.095–4.08)
Use of ciprofloxacin	−0.91659 ± 1.0877	0.3994	0.399 (0.044–3.34)
Use of moxifloxacin	−0.5060 ± 2.1187	0.8112	0.603 (0.009–4.164)

Use of norfloxacin	Reference	Reference	Reference
Use of ofloxacin	14.17927 ± 902.301	0.9875	1,438,735 (4.23e‐55‐NA)
Number of co‐morbidities	0.1419 ± 0.169	0.4003	1.15 (0.835–1.62)
Intercept	144.508 ± 143.51	0.3139	5.74e+62 (4.79e‐60 2.91e+186)

Note

Negative coefficient estimates indicate inverse relationship between predictors and outcomes.

Abbreviations: CI, confidence interval; OR, odds ratio.

**TABLE 4:** Analysis of patient characteristics’ association with bilateral tendon ruptures from the 145 claimants who received compensation based on the Finnish Pharmaceutical Insurance Pool's pharmaceutical injury claims 2002–2012

Variable	Estimate ± standard error	*p*‐value	OR (95% CI)
AE year	−0.1613 ± 0.098	0.09971	0.851 (0.694–1.023)
Male gender	0.3561 ± 0.6171	0.56396	1.4277 (0.4505–5.2147)
Age	0.0253 ± 0.0271	0.34990	1.02567 (0.9749–1.0855)
Use of oral steroids	1.38169 ± 0.5217	0.00808	3.98 (1.47–11.68)
Use of levofloxacin	15.2212 ± 1423.56	0.99147	4,078,675 (6.291e‐26 5.461e+180)
Use of ciprofloxacin	15.211 ± 1423.56	0.99147	403,8437 (4.353e‐29‐NA)
Use of moxifloxacin	−0.82358 ± 2724.65	0.99976	0.439 (3.028e‐46 ‐NA)

Use of norfloxacin	Reference	Reference	Reference
Use of ofloxacin	−1.49990 ± 2913.87	0.99959	0.223 (2.28e‐49‐NA
Number of co‐morbidities	0.09090 ± 0.211	0.66723	1.095 (0.71–1.65)
Intercept	303.91848 ± 1437.06	0.83251	9.775e+131 (NA–1.9461e+167)

Note

Negative coefficient estimates indicate inverse relationship between predictors and outcomes.

Abbreviations: CI, confidence interval; OR, odds ratio.

## 4 DISCUSSION

Our study found that the 145 claimants with tendon injuries had an average of 14,800€ direct costs in addition to the indirect costs that were estimated to be 9077€ for employed claimants. Fifty‐one percent were hospitalized, with an average duration of 21 hospital days. Hospitalization was the costliest form of health service use amounting to an average of 9915€ per hospital episode. Advancing age and having co‐morbidities made claimants more likely to have longer hospitalizations or less likely to avoid hospitalization altogether. The prescribed FQ, use of oral corticosteroids, or the claimant's gender had no significant influence on hospitalization.

The number of FQ prescriptions has almost halved during the 2010s with all antibiotic prescribing steadily declining in Finland, and during the past few years, FQs have comprised approximately 5% of antibiotic prescriptions.^26^ Tendon injuries were first connected to norfloxacin in 1983 and have since been associated with all FQs currently on the market.^13^, ^27^ Unlike many other drug‐related AEs, FQ‐related tendon injuries are generally easy to detect, and the causal effect between drug and AE is often explicit. Therefore, the pharmaceutical insurance claims were mostly compensated and only rejected if the claimant did not provide timely documentation or if the resulted tendon injury was regarded as tolerable in relation to the severity of the treated infection. The underreporting of AEs poses a significant problem for research relating to AEs and pharmaceutical injuries. A larger sample size would fortify the evidence and provide more robust estimates. Since 2012, the handling of pharmaceutical injury insurance claims was taken over by the Finnish Co‐operative for Pharmaceutical Injury Indemnities from the Insurance Pool, which was in force since 1984. Approximately 200 pharmaceutical injury insurance claims are filed annually, which may suggest that many injuries remain both unidentified and uncompensated, and that the compensation system continues to be relatively unknown. According to a systematic review published in 2006, 94% of all AEs possibly elude reporting.^28^ Consequently, the 160 FQ‐related tendon injury claims filed to Insurance Pool between 2002 and 2012 were most likely only a small fraction of the actual amount^29^ and to make assumptions about the entire costs associated with FQ‐related tendon injury costs based on this data would be misguided. Similar no‐fault pharmaceutical injury insurances are in use for example in Sweden, Norway, and Denmark. Unit costs relating to healthcare systems are country‐specific and for this reason, costs presented in the results are directly applicable only to Finland. However, health service use, such as the number of clinic visits and hospital days are can be transferred, particularly if the study population is comparable.

As studies have shown drug‐related AEs to be a major clinical and economic problem,^30^, ^31^ there is a need for published literature regarding societal costs associated with drug‐related AEs. Lost productivity was only compensated to six claimants, and there was substantial variation and missing documentation of earnings. Therefore, average earnings from Finland were chosen to provide a more realistic average estimate of indirect costs. However, the direct costs in the study were true societal costs based on real‐world evidence. Economic evaluations of interventions have a significant role when implementing products and practices to healthcare systems. Methods such as cost‐benefit, cost‐effectiveness, and cost‐utility analyses have become standards for prioritizing and comparing interventions, with the latter two capturing the element of patient reported quality of life. However, cost‐of illness studies, such as this, benefit decision‐making from an entirely different perspective, namely, the economic burden to the society. Incapacity compensations offer a glimpse of the value of health‐related quality of life lost due to an AE, nonetheless, they cannot be used as a proxy.

At present, clinicians are more likely to avoid prescribing FQs to risk groups than in 2012, due to EMA and FDA recommendations, as well as improved general awareness of the AEs. In addition, treatment practices of tendon injuries have changed slightly toward favoring conservative treatment.^32^ Yet, a recent study by Westin et al.^33^ has found patients treated with surgery to have a consistently better health‐related quality of life compared with those treated conservatively during a one‐year follow‐up. However, it appears that treatment choice does not have a major impact on the costs because both immobilization and surgery required an equally long hospital stay in the patients who were compensated. In contrast to other serious AEs associated with FQs, such as cardiac toxicity,^34^ tendon injuries are not a direct cause of mortality. However, research has shown that traumatic injuries are associated with significantly higher morbidity and mortality in elderly patients than in their younger counterparts.^35^ The death of eight claimants during the compensation claim process affected the amount of injury compensations. It is a common practice for insurance companies to hold back the total compensation until the level of permanent disability has been evaluated. In such circumstances, the cost of a tendon injury episode will be underestimated.

Although the number of levofloxacin‐based claims might give the impression that its tendency to cause tendon injuries exceeds that of other FQs, this is likely not the case. Forty‐five percent of the claimants had previously been diagnosed with chronic lung diseases, which made them more susceptible to respiratory infections that were often treated with levofloxacin. Patients with chronic lung disease are frequently treated with inhaled glucocorticoids, as well as systemic glucocorticoids, which strongly increase the risk of FQ‐associated tendon injuries. Moreover, it is possible that some of these patients had received fluoroquinolones on several occasions. The risk of tendon injuries appears to rise with an increasing number of fluoroquinolone prescriptions and with increasing cumulative dose^6^; the accumulated risk has been estimated to be approximately 6% with each additional day exposed to FQs.^36^ Thus, we believe that levofloxacin was overrepresented in the present study sample, because it was often used in high‐risk patients.

EMA has recommended restricting FQ use in infections that are not severe, as a first‐line treatment and to avoid prescribing to high‐risk groups, such as those using corticosteroids.^3^ With current recommendations, it may be possible to avoid some of the tendon injuries, but it is likely that in some cases there are no good alternatives to FQs. Moreover, some of the tendon injuries occur to patients who have no known risk factors, as also seen in our study, and, therefore, some of the cases remain unavoidable also with current knowledge.

Medical staff needs to stay wary of the risks in order to prevent and reduce fluoroquinolone‐related tendon injuries, in addition to health service use and costs associated with them. However, due to the growing amount of antimicrobial‐resistant bacteria, the rapid decline of effective antimicrobials, and alarmingly small number of new discoveries, AEs and resulting costs might have to be increasingly tolerated in the future, in order to combat bacteria. Additionally, decision makers need to be aware of the economic burden of AEs from a societal perspective as its extent is often underestimated.

## DISCLOSURE

The authors of this manuscript have no conflicts of interest to declare.

## AUTHOR CONTRIBUTIONS

All persons who meet authorship criteria are listed as authors. All authors took part in conceptualizing the idea, designing the study, analyzing the data and writing the manuscript. All authors have seen and approved the final version of the manuscript.

## ETHICAL APPROVAL

This is a retrospective observational study with register data, and neither an ethics approval nor patient consent was required.

## Data Availability

The data that support the findings of this study are available on request from the corresponding author. The data are not publicly available due to privacy restrictions.
